# A Clinical, Histopathological and Immunohistochemical Approach to the Bewildering Diagnosis of Keratoacanthoma

**Published:** 2014-09

**Authors:** Massoumeh Zargaran, Fahimeh Baghaei

**Affiliations:** a Dental Research Center and Dept. of Oral and Maxillofacial Pathology, School of Dentistry, Hamadan University of Medical Sciences, Hamadan, Iran.; b Dept. of Oral and Maxillofacial Pathology, School of Dentistry, Hamadan University of Medical Sciences, Hamadan, Iran.

**Keywords:** Immunohistochemistry, Keratoacanthoma, KA, CD30

## Abstract

Keratoacanthoma (KA) is a comparatively common low-grade tumor that initiates in the pilo-sebaceous glands and pathologically mimics squamous cell carcinoma (SCC). Essentially, strong debates confirm classifying keratoacanthoma as a variant of invasive SCC. The clinical behavior of KA is hardly predictable and the differential diagnosis of keratoacanthoma and other conditions with keratoacanthoma-like pseudocarcinomatous epithelial hyperplasia is challenging, both clinically and histopathologically. This article aims to illustrate and explicate some of these complicated issues by presenting two cases of KA and a relevant review of literature. It also targets the clinical, histopathologic, and immuno-histochemical features of these two cases.

Both presented lesions of this study had appeared on the vermilion border of the lower lip and no vascular or perineural invasion was observed. The results of the immuno-histochemical survey, particularly in staining with marker CD30, confirmed the differential diagnosis of keratoacanthoma from keratoacanthoma-like pseudocarcinomatous proliferations which was consequent to the CD30^+^ lymphoid infiltration.

Histopathological and immunohistochemical investigation is necessary to disprove the invasive biologic behavior of keratoacanthoma and also to refute all conditions with keratoacanthoma-like pseudocarcinomatous epithelial hyperplasia.

## Introduction

Keratoacanthoma (KA) is defined as a benign keratinocytic neoplasm which arises from human’s hair follicle [1-3]. It is usually detected as a single dome-shaped nodule with a central crater filled with keratin [2-5]. It is a fast-growing lesion which regresses and confines spontaneously [5]. KA is reported to be a lesion which rapidly grows during 6 to 8 weeks [2, 4]. The growth phase is followed by a growth-stop period and then by a four- to six-week period of impulsive regression. After the lesion is resolved, an atrophic and hypopigmented scar is left [2, 4].

The disease is frequently seen in the elderly people with light skins and in the areas which are exposed to sun, especially lips, the vermilion border of the lips, cheeks, nose and the back of the hands. The lesion has the same male and female predilection with a slightly more tendency to male individuals [1, 4-6]. This report presents a clinical, histopathological and immuno- histochemical study of two cases of KA of the lower lip, referred to the oral pathology department of Hamadan Dental School, Iran. Besides, the clinicopathological and immunohistochemical characteristics of the lesions are elucidated.

## Case Report

The current study investigated only two cases of KA which were available in the archives of the department of oral pathology, faculty of dentistry, Hamadan University of Medical Science, Iran.

 Clinical and microscopic information of the lesions was obtained by scrutinizing the patients’ dental documents and the available histopathological slides available in the patients’ archives. The best and the most pertinent paraffin blocks were selected to perform the subsequent immunohistochemical staining. 

## Clinical Findings

Table 1 illustrates the specific information regarding the cases of keratoacanthoma, scrutinized in this study.

**Table 1:** Summarized information of cases (Sex, Age, Duration, Location, Size and Clinical manifestations)

**Case**	**Sex**	**Age**	**Time from appearance to surgery**	**Location**	**Size**	**Clinical manifestations**
Case 1	Male	54	2 months	lower lip, vermilion border, right side	8 × 5mm	Sessile red-brownish nodule with superficial ulceration and partial crust covering
Case 2	Female	60	12 months	lower lip, vermilion border, right side	13×11mm	Dome-shaped brownish-black nodule with a necrotic crust

The only symptom accompanied by the lesions was reported to be a slight pain in the lesion site in the first case. The significant finding, considering the same case, was a palpable lymph node under the chin which was removed with the lesion during a surgery. This finding instigated the diagnosis of the disease to be squamous cell carcinoma (SCC) and subsequently KA in differential diagnoses. 

Both cases proposed negative history of medication consumption, systemic diseases, malignancy, smoking and presence of any other lesion. 

The surgical excision was performed as the treatment of both cases; each removed full thickness with a safe margin (1 cm and 0.5 cm safe margin for the first and the second case respectively).

## Histopathological Findings

The microscopic examination of the two cases displayed hyperplasic squamous epithelium with a central crater-like depression extended into the underlying connective tissue. The papillary surface of the lesion was covered with a thick layer of parakeratin having central plugging. 

**Figure 1:**
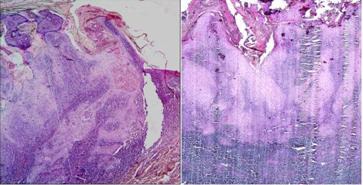
Histopathological feature of KA, H&E staining

The accumulation of keratin, configuring as keratin pearls, was observed and individual cell keratinization could sporadically be detected; mostly in the upper parts of the lesion. The proliferation of the epithelial cells in the base of lesion had protracted into the underlying fibro-connective tissue in ritual of irregular aggregates. However, it was not spread into the muscles and sweat glands. The superficial epithelium on the lateral border of the tumor appeared to be normal. There was an unblemished acute angle between the lesion and the overlying epithelium in the outer rims of the central crater of the lesion (Figure 1).

In the first case, the epithelial cells of the lesion presented slight cellular atypia. However, the second case displayed slight cellular pleomorphism and more keratin pearls. 

In the fibro-connective tissue of both cases, the heavy infiltration of the chronic inflammatory cells with predominance of lymphocytes could be observed, exclusively near the base of the lesion (Figure 1).

## Immunohistochemistry

Sections with 4 μm thickness were cut from paraffin- embedded tissue blocks. The antibodies against cytokeratin (CK); epithelial membrane antigen (EMA), CD30, CD34 and S100 were applied to the sections according to their manufacturers’ instruction. The present study evaluated the expression of CD30 in two methods:

1. Quantitative; which attains the labeling index (LI) by counting the number of positively-stained cells per 1000 cells.
2. Semi-quantitative; which is concerns the criteria anticipated by Fernandez-Flores [7].

The percentage of positive cells was scored on a scale of 0 to 4; 0 for absence of CD30+ cells, 1 for occasional finding of CD30+ cells, 2 for detecting the CD30+ cells more than occasional but still non-grouped, 3 for presence of CD30+ cells in groups containing 3 or less cells, 4 for observing the CD30+ cells in groups containing more than 3 cells [7].

## Immunohistochemical Findings

**Figure 2:**
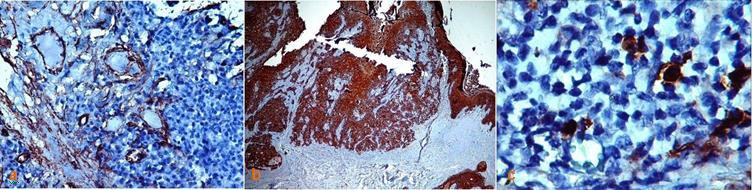
a:  CD34+ stained vessels without any invasion of tumoral cells  b: Diffuse immunostaining of epithelial cells with CK, but no staining was detected among chronic inflammatory cells  c: Immunostaining  reveals a few CD30+ cells

The immuno-histochemical staining of the two KA cases with CD34 and S100 revealed that the vessels were positively stained with CD34 marker. However, no evidence of tumoral-cell invasion was identified in the positively stained vessels (Figure 2a). Nerve fibers were positively stained with S100; likewise, no evidence of perineural invasion of the stained nerves was perceived in these samples. Moreover, staining was performed by employing CD30, CK, and EMA markers to scrutinize the nature of the possible atypical cells in the dermal inflammatory infiltration.

The cytoplasm and the cytoplasmic membrane exhibited positive CK and EMA staining in epithelial cells, but the antigens were negatively stained in the region of dermal lymphocytic infiltrations (Figure 2b).

A few positively CD30 stained cells were extensively dispersed in the inflammatory infiltration zone of the lesions (Figure 2c).

The LI was yielded as 2.1% for the first case and 0.4% for the second case. Hence, based on the criteria proposed by Fernandez-Flores [7]; positive cellular staining level was determined as 1.

The CK, EMA and CD30 immuno-staining result for the resected lymph node was negative. 

## Discussion and Literature Review

Kerathoacanthoma was first described by Jonathon Hutchison in 1889 as a distinct lesion with a crater-like facial ulcer [8]. This lesion; which most commonly involves the face and hands, is a rapidly-growing cutaneous tumor with atypical histopathological manifestations that resembles the squamous cell carcinoma (SCC). It leaves an atrophic scar when resolves [4-5].

The disease is more common in male individuals and the research has revealed 80% of the patients were above 40 years old whilst the pick incidence of the disease occurred in people with 45 to 69 years old [5].

The anticipated etiologic factors for the lesion include sunlight (ultraviolet ray), chemical carcinogens, positive history of trauma, genetic factors, viruses, chemotherapy, immunological factors and cigarette smoking [4-5].

The current report represented two patients with solitary KA in the abovementioned age range. In addition, over exposure to sun rays emerged as the most probable etiologic factor in both cases.

KA is considered as a benign tumor of skin; a prototype of pseudo-malignant skin tumors. It is also believed that this lesion is a malignant neoplasm and should be deliberated as an abnormal variety of SCC of the skin [1]. This assertion is made since KA, unlike SCC, is a lesion with spontaneous healing [9]. However, the tumor shows unpredictable and aggressive growth in some instances [6] and induces local destructions [2]. Some cases of KA were reported to be metastasized to other organs [2-3].

Therefore, the question may arise that whether the keratoacanthoma is a distinct lesion or it is essentially a type of SCC [3, 9-10].

Fortunately, when a typical clinical history is available, the morphologic features and the growth pattern of KA are adequate to diagnose most of the patients. Keratoacanthoma can display an exophyticendophytic pattern of growth; a protuberant lesion with a depressed central crater filled with keratin, while SCC of the skin, routinely displays a form of endophytic growth. Histopathologically, KA has some explicit characteristics [5]. The superficial epithelium on the lateral border of the tumor appears normal but at the lip of the central crater of the lesion, a definite acute angle between the lesion and the overlying epithelium is spotted [11].

The crater is filled with keratin and the epithelial cells in the base of lesion proliferate downward and generally induce a substantial chronic inflammatory response. Dyskeratosis can be observed comparable to well-differentiated SCC, whether as the individual cell keratinization or as the keratin pearls [11].

The architecture of the tumor is more important than its cytological features in diagnostic procedures [11] since the lesion may exhibit the microscopic features of SCC such as infiltration and cellular atypia [3].

In the histopathologic surveys of the current study, both cases demonstrated typical histopathologic patterns of KA, although, slight cellular pleomorphism were identified in the second case. 

A palpable lymph node was detected in the first case, removed with the lesion in the surgical therapy. To reject the probability of malignancy or the existence of any metastasis in this case, immunohistochemistry staining EMA, CK, and CD30 markers were employed. Since the results were negative, it looks as if the lymphadenopathy was due to the inflammatory reactions rather than malignancy or else, nodal metastasis.

Perineural invasion has been reported in 0.5%-36% of SCCs of the head and neck skin. This is uncommon (1-4 %) in keratoacanthoma [12]; therefore, the condition can easily be ignored by the pathologists. Perineural invasion in KA has been reported in some studies [13-15] and the related complications may differ broadly from case to case. The complications are reported to be the extension of the lesion into the facial mimic muscles [13, 15], recurrence of the lesions [12-14, 16], growing into the cranial nerves [16], invasion to the cavernous sinus [17] and metastasis to the parathyroid gland and also to the local and auxiliary lymph nodes [18].

Therefore, it has been advocated that the perineural invasion may indicate the potential aggressive growth of the KA in the head and neck region; which clinically appears as disrupted responsiveness in association with neuropathic pain [12].

In the present study, histopathological surveys revealed no evidence of perineural invasion. The perineural invasion is missed by pathologists because of the dense infiltration of inflammatory cells. To avoid this missing, particularly in the first patient who had complaint of pain, both samples were stained with S100 marker to show the nerves and to find out if the tumoral cells were present around the neural bundles. The result for the perineural invasion was negative. 

Vascular invasion is another symptom of tumoral invasions and some pathologists consider KA as a type of well differentiated SCC when this feature is observed [19].

Both lesions of the present study were examined concerning the vascular invasion. We employed CD34 marker for a careful survey of vessels and the result was negative.

Although Kurien et al. [19], Janecka et al. [20] and Calonje and Jones [21] have confirmed the benign clinical behavior of KA even in the presence of vascular invasion. They have reported the invasive histopathological characteristic was not consistent with the benign clinical behavior of the lesion and could not be considered as an evidence of malignant transformation and metastatic potential of KA. Thus, this issue can confirm the significance of clinico-pathological surveys in the process of lesion diagnosis [19-21].

When the KA of the skin is examined, the CD30^+ ^lymphoproliterative disorders such as anaplastic large cell lymphoma (ALCL) and lymphomatoid papulosis (LyP) should be deliberated as the differential diagnoses [22-23].

The CD30+ atypical lymphocytes, present in these types of lesions, may induce epidermal proliferation by means of production of cytokines, epidermal growth factor (EGF) molecules or other substances [7]. This may appear as pseudocarcinomatous hyperplasia, resembling KA under the microscopic examinations [24, 7].

Regarding the pseudocarcinomatous proliferations related/secondary to the CD30^+^ lymphoid infiltration, specific terms are elaborated such as primary cutaneous CD30^+^ anaplastic large cell lymphoma mimicking keratoacanthoma [25] or CD30 anaplastic large cell lymphoma with keratoacanthoma-like pseudocarcinomatous hyperplasia. The aforementioned lesions can incorrectly be diagnosed as KA [24].

Such lesions, even in the presence of CD30^+^, can carry on some challenges in the diagnosis of KA. In H&E staining, the diagnosis of CD30^+^ lymphocytes; that appear as the large *epithelioid cells* in the inflammatory infiltration area of the lesions, is very difficult since they are obscured simply by a massive infiltration of small lymphocytes, neutrophils, eosinophils, and histiocytes [24].

Immunohistochemistry staining with CD30^+ ^marker can confirm the presence of these atypical lymphocytes. Although CD30^+ ^cells can be assumed as the diagnostic attribute for CD30^+ ^lymphoproliterative disorders, they are not pathognomonic [26]. Moreover, the neoplastic epithelial proliferations such as KA may subsequently induce CD30^+^ lymphocytes [23, 27]. Some studies reported the CD30^+^ cells as a common constituent in the inflammatory infiltration of KA [7, 28]. The quantitative data offered by these studies would probably be helpful in the differential diagnosis of these two groups of lesions, particularly when the patients do not suffer from any skin or hematologic disorders. For instance, regarding the quantitative criterion in the diagnosis of CD30+ large cell lymphoma; more than 70% of the cells of the lesion should be stained for CD30. Likewise, the expression of CD30 by the atypical cells in lymphomatoid papulosis (LyP) should range from 25% to more than 90% [24, 7].

However, in the case of KA, especially when it is in regression period, the expression ensues at a very low level. The mean percentage of the CD30 expression for 18 cases of KA has been testified to be 2.89% in the study of Fernandez-Flores [7]. In that study, the maximum level of CD30 expression was reported 10.54% and the minimum level was stated to be 0.24%; related to the case of KA in regression [7]. In another study, enrolled by the same researcher, the mean percentage of CD30 expression for KA in regression has been reported to be 0.58% [28].

Since Cepeda et al. have also reported 4.8% of benign skin lesions with inflammatory infiltration [29]; it is proposed that a number of CD30+ cells of KA are similar to other benign inflammatory infiltrates [7]. In the current study, the number of CD30^+^ cells was 2.1% for the first case and 0.4% for the second case. This is in agreement with the above-mentioned studies [7, 28-29]. However, it seems that the difference in the expressions between two presented cases was associated with the difference in time taken by the lesions to emerge.

KA is a skin neoplasm with a rapid growth whose natural course is usually accompanied with spontaneous regression [6]. Various treatment methods have been recommended for KA [2, 5-6, 30] but because of the biologic behavior of the lesion and the possible spontaneous regression of the lesion, attentive follow up is likely to replace the conventional treatment [2].

KAs are frequently treated in the initial phases due to different reasons [1-3, 31] and a few lesions have been reported to be resolved with spontaneous regression (the gold-standard for the diagnosis) without any therapy [4].

Some of the proposed reasons for the treatment of KA are illustrated in Table 2.

**Table 2:** Some of the anticipated reasons for treatment of keratoacanthoma

**The anticipated reasons for treatment of keratoacanthoma**
Minimizing the scars left after the regression of the lesion [2] Local destruction which follows the rapid growth of the lesion and metastasizes to other organs reported in some cases [2-3] The tendency of KA to appear on the face and the probability of destruction of a large area of the tissue due to the ulceration and secondary infection [31] The unpredictable final size of the lesion [31] The long period of time required for the regression of the lesion that may even take more than one year (9 to 12 month at least) [1] The unpredictable course of some these tumors, exhibiting aggressive behavior like SCC [6] The initial worrisome characteristic of the lesion, the dilemma to reach a definite diagnosis and the concerns for the clinical differentiation of the lesion from SCC or other aggressive lesions in its growth phase [2, 4]

The selected treatment for the lesion is complete surgical excision [2] to which the solitary KAs respond well [5]. Some advantages and disadvantages of this procedure include rapid treatment, the ability to examine the lesion histopathologically, to prevent local invasion and metastasis and to minimize the scarring.

However, the surgical procedure may be destructive and when the lesions are located on the esthetically or functionally important regions, the treatment could be unacceptable [2].

Other treatment modalities such as electro-surgery, cryo-surgery, laser-surgery, curettage, radiotherapy, systemic chemotherapy, topical chemotherapy (intra-lesion injection) and photodynamic therapy have been also practiced [2, 5-6, 30]. These different treatment modalities may convey different results whilst appended with some limitations and side effects [2, 5-6].

Surgical interventions such as laser, electro and cryosurgery may develop esthetic defects or functional disabilities. Moreover, these approaches may interrupt the histopathological endorsement of the clinical diagnosis [2]. Radiotherapy can be effective in the cases of recurrence or reappearance of the lesions after the surgery or the patients in whom the resection would bring unacceptable results and cosmetic disfigurements [30], although it is not a suitable approach for treating young adult patients. The treatment is expensive and difficult to be performed since it compels frequent visits at hospital. Skin atrophy, radiodermatitis and increased carcinogenic potential are stated to be the other side effects of the treatment [2, 6].

Treating with intra-lesion injections and topical agents has also been described to be imperative but they are sometimes accompanied by some side effects.

The intra-lesion injection of methotrexate can intricate pancytopenia, intra-lesion injection of 5-fluorouracil would elaborate local pain and thus requires anesthesia and finally, the application of Imiquimod cream can develop immunological reaction such as burning sensation, erythema and erosion [2, 6].

Both cases of keratoacanthoma, described in this report, endured complete surgical excision. In the first case, the reason for the surgery might have been the concerns for SCC and its feasible metastasis in the presence of palpable lymph node. The absence of regression of the lesion after one year would likely be the reason of surgery for the second case. Surgery is the recommended therapy for suspected solitary KAs when they exhibit abnormal growth pattern after 4-6 weeks [4].

## Conclusion

The diagnosis and treatment of KA is a challenging task, hence, careful clinical, histopathologic and immuno-histochemical examinations of the lesion are the prerequisites. They help for an appropriate diagnosis and may signify an effective treatment by determining the exact biologic behavior of the lesion and refuting the diagnosis of other lesions exhibiting with keratoacanthoma-like pseudocarcinomatous epithelial hyperplasia. 
